# A Case of Systemic Lupus Erythematosus Complicated by Secondary Evans Syndrome

**DOI:** 10.7759/cureus.80959

**Published:** 2025-03-21

**Authors:** Madison Drallmeier, Meghan Grossmann, Alexis Haftka-George

**Affiliations:** 1 Internal Medicine, Henry Ford Health System, Detroit, USA; 2 Internal Medicine, Wayne State University, Detroit Medical Center, Detroit, USA

**Keywords:** autoimmune hemolytic anemia, autoimmune neutropenia, evans syndrome, immune thrombocytopenia, secondary evans syndrome, systemic lupus erythematosus

## Abstract

Evans syndrome (ES) is a condition that describes the development of multiple cytopenias, including autoimmune hemolytic anemia (AIHA), immune thrombocytopenia (ITP), and autoimmune neutropenia (AIN). ES can be idiopathic or caused by an underlying condition, known as secondary ES. While secondary ES is associated with increased morbidity and mortality, any diagnosis of ES confers a poor prognosis. In this case report, we describe a young male patient diagnosed with systemic lupus erythematosus (SLE) and secondary ES that was complicated by multiple relapses and subsequent infections, bleeding events, and thrombotic events that ultimately led to the passing of the patient.

## Introduction

Evans Syndrome (ES) is an autoimmune condition that was first described in 1951 as a condition in which an individual develops multiple cytopenias: autoimmune hemolytic anemia (AIHA), immune thrombocytopenia (ITP), and less often autoimmune neutropenia (AIN) [1]. It has been further classified as primary ES or secondary ES, which helps differentiate if the condition is idiopathic or the result of another disease. Conditions associated with secondary ES include lymphoproliferative disorders, such as non-Hodgkin lymphoma (NHL) and chronic lymphocytic leukemia (CLL); viral illnesses, such as human immunodeficiency virus (HIV), Hepatitis B (HBV), and Hepatitis C (HCV); primary immunodeficiencies, such as common variable immunodeficiency (CVID) and autoimmune lymphoproliferative syndrome (ALPS); and autoimmune diseases, such as systemic lupus erythematosus (SLE), rheumatoid arthritis (RA), antiphospholipid syndrome (APS), and Sjogren’s [2]. The few large studies that have been completed regarding the epidemiology of ES have demonstrated that 21-50% of cases are due to secondary causes such as those noted above [3-5]. Unfortunately, these studies have also shown that ES is challenging to treat and can be complicated by thrombosis, infections, and bleeding events that can ultimately result in death [3-5]. In our case, we demonstrate the difficulties faced in treating a patient with ES and the associated complications.

## Case presentation

An 18-year-old male with no past medical history presented to the emergency department for several days of generalized malaise and sore throat. He had been examined by his pediatrician days earlier, who diagnosed him with streptococcal pharyngitis and prescribed him antibiotics. However, he failed to improve. His physical exam was positive for a low-grade fever, a 3-millimeter erythematous non-bleeding erosive mucocutaneous lesion in his left nostril, an erythematous pharynx, and an anxious disposition. His initial laboratory workup revealed significant laboratory abnormalities, including elevated creatinine, hyperkalemia, and elevated liver enzymes (Table 1).

**Table 1:** Complete metabolic panel ALT: alanine aminotransferase; SGPT: serum glutamic pyruvic transaminase; AST: aspartate aminotransferase; SGOT: serum glutamic oxaloacetic transaminase; ALP: alkaline phosphatase

	Result	Reference Range
Sodium	127	135-145 mmol/L
Potassium	6.2	3.5-5.0 mmol/L
Chloride	102	98-111 mmol/L
CO2	21	21-35 mmol/L
Blood Urine Nitrogen	66	10-25 mg/dL
Creatinine	1.91	<1.28 mg/dL
ALT/SGPT	174	<52 IU/L
AST/SGOT	340	<35 IU/L
Albumin	1.7	3.7-4.8 g/dL
Bilirubin, Total	0.7	<1.2 mg/dL
Bilirubin, Direct	0.3	0-0.3 mg/dL
ALP	102	50-180 IU/L

Further investigation revealed microcytic anemia, severe thrombocytopenia, decreased haptoglobin, increased lactate dehydrogenase and uric acid, and a positive direct antiglobulin test (DAT). Together, these findings suggested an autoimmune-mediated hemolytic process (Table 2).

**Table 2:** Complete blood count and hemolysis labs MCV: mean corpuscular volume; MCH: mean corpuscular hemoglobin; MCHC: mean corpuscular hemoglobin concentration; RDW: red cell distribution width; DAT: direct antiglobulin test

	Result	Reference Range
White Blood Count	6.6	3.8-10.6 K/uL
Red Blood Count	3.78	4.4- 6.0 M/uL
Hemoglobin	9.6	13.5-17 g/dL
Hematocrit	28.80%	41-53%
MCV	76.3	80-100 fl
MCH	25.3	26-34 pg
MCHC	33.2	31-37 g/dL
RDW	14.20%	<14.5%
Platelet Count	17	150-450 K/uL
Haptoglobin	<30	30-200 mg/dL
Lactate Dehydrogenase	464	<250 IU/L
Uric Acid	8.5	2.5-7.5 mg/dL
DAT	Positive	Negative

Finally, a peripheral smear was obtained given the high concern for thrombotic thrombocytopenic purpura (TTP), and showed microcytic anemia with thrombocytopenia but was negative for schistocytes.

**Figure 1:**
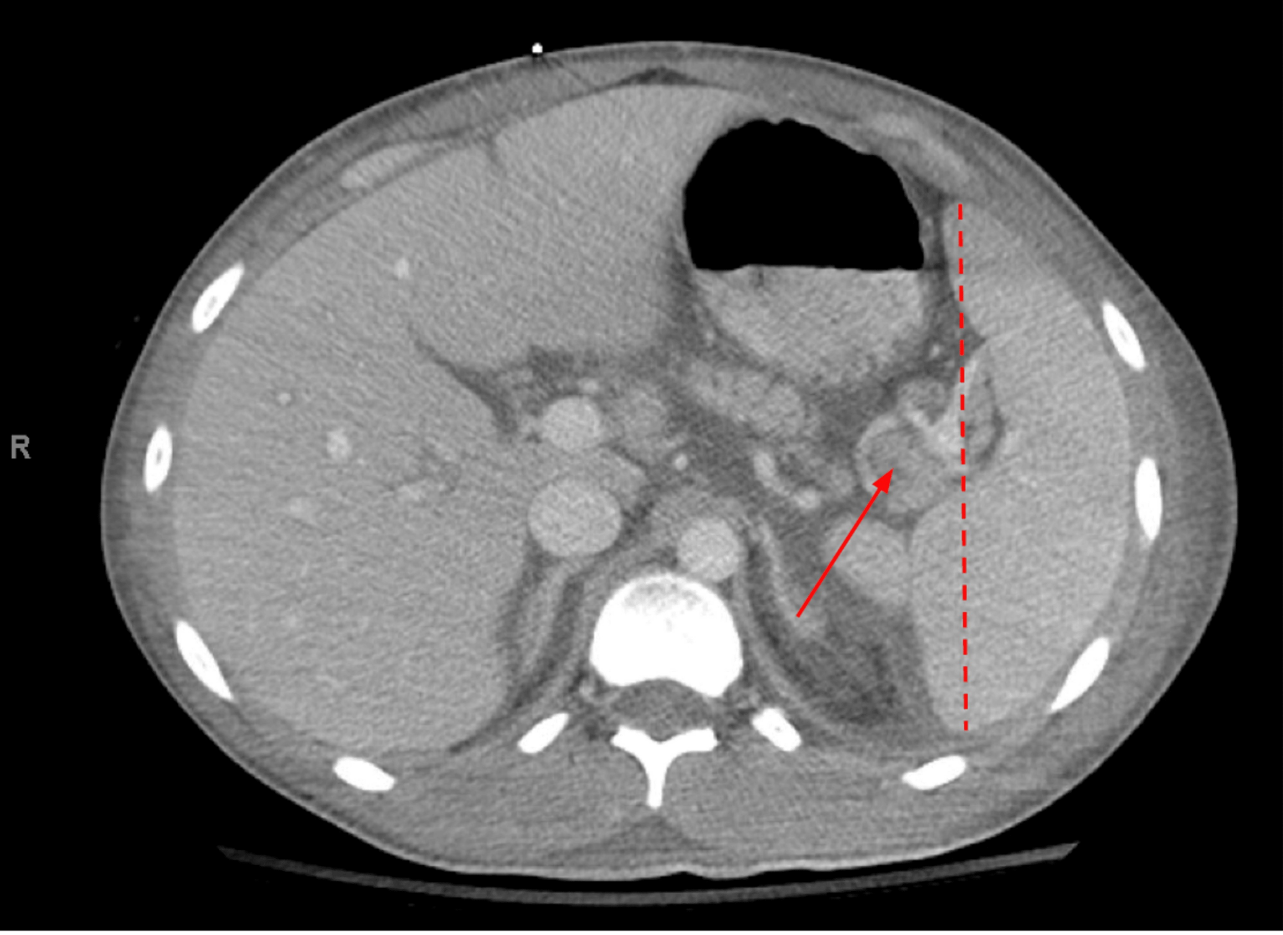
Computed tomography of the chest, abdomen, and pelvis: cross-section The red dashed line represents the length of the spleen, which is mildly enlarged at 14.3 centimeters (normal length <13 centimeters); the red arrow points to an enlarged lymph node within the splenic hilum, measuring near 10 millimeters, which is favored to be reactive.

A broad workup was completed to elucidate the etiology of the patient’s hemolytic anemia and thrombocytopenia. Infectious workup, including Ebstein Barr virus (EBV), cytomegalovirus (CMV), HIV, HBV, HCV, and parvovirus B19 were negative. However, the patient’s blood cultures were positive for methicillin-susceptible Staphylococcus aureus (MSSA), and the patient was started on intravenous cefazolin. The patient was also evaluated for malignancy with a computed tomography (CT) scan. His CT of the chest, abdomen, and pelvis revealed splenomegaly with mildly enlarged lymph nodes present in the adjacent splenic hilum, which were favored to be reactive (Figure 1).

Serum and urine protein electrophoresis were negative. Finally, the patient was tested for autoimmune processes, which were significant for a positive antinuclear antigen (ANA; ratio >1:1280; homogenous pattern), positive double-stranded deoxyribonucleic acid (dsDNA; ratio >1:320), and decreased complement (C3 and C4) levels. Other autoantibodies, such as rheumatoid factor, Ro/La, smooth muscle antibody, liver-kidney microsomal antibody, beta-2 glycoprotein, lupus anticoagulant, and anticardiolipin, were negative (Table 3).

**Table 3:** Serologic studies ANA: anti-nuclear antibody; ds DNA, IFA: double-stranded deoxyribonucleic acid, immunofluorescence assay; C3: Complement 3; C4: Complement 4; PTT-LA screen: lupus anticoagulant testing

	Result	Reference Range
ANA	Positive	Negative
ANA Pattern	Homogenous	-
ANA Titer 1	>1: 1280	<1:80 titer
ds DNA Ab, IFA	Positive	Negative
ds DNA Ab, Titer	>1: 320	<1:10 titer
C3	27	90-230 mg/dL
C4	<8	10-51 mg/dL
Cardiolipin Ab, IgG	4.2	<10 GPL
B2 Glycoprotein IgG	4.9	<7 U/mL
PTT- LA Screen	36.7	30.3-43.2 seconds
Rheumatoid Factor	<10	<14 IU/mL
SS A/Ro Ab	<0.2	<1.0 Elisa Units
SS B/La Ab	<0.2	<1.0 Elisa Units
Smooth Muscle Ab, IgG	10	<20 units
Liver-Kidney Microsomal Ab	1.7	<20 units

Based on his clinical and immunological findings, including fever, positive ANA, elevated dsDNA, low complement, thrombocytopenia, and autoimmune hemolysis, the patient met the diagnostic criteria for SLE with concomitant Evans syndrome. The patient was started on methylprednisolone (250 milligrams/day) for a total of 3 days, followed by an oral prednisone taper (1 milligram/kilogram/daily with a 10-milligram decrease every week) as well as hydroxychloroquine 300 milligrams daily for the treatment of his SLE. He was recommended to undergo a renal biopsy for an evaluation of lupus nephritis, but the patient declined and requested it to be done as an outpatient. While many of his laboratory studies, such as renal function and liver studies, improved throughout his hospitalization with treatment of his SLE, he continued to have significant cytopenias. He was started on rituximab therapy since his ES was considered refractory to first-line treatment. Unfortunately, he developed angioedema after the first dose of rituximab, and it was discontinued. He was recommended to undergo inpatient de-sensitization of rituximab, however, he opted not to pursue this option and was discharged from the hospital.

As the patient continued outpatient follow-up with multiple sub-specialties, he was noted to have poor adherence to multiple lines of medical therapy. Although he was encouraged to continue hydroxychloroquine (dose adjusted to 200 milligrams daily) for treatment of his SLE, the patient expressed concerns about possible side effects and opted against treatment. He also prematurely discontinued his prednisone taper due to weight gain and insomnia. His nephrologist also identified a progressive increase in proteinuria, which peaked at 4.7 grams/gram, as well as worsening hypertension, and he was started on losartan 25 milligrams daily. He also was not adherent with losartan and his outpatient blood pressures were as high as systolic pressures into the 180s. Three months following his initial hospitalization, the patient developed decreased platelet counts and hemoglobin levels as well as a significant increase in his creatinine. His rheumatologist organized for the patient to receive intravenous methylprednisolone as an outpatient, however, he was not able to tolerate his first dose and was admitted to the hospital. He completed a 3-day course of methylprednisolone (250 milligrams/day) and transitioned to an oral prednisone taper (1 milligram/kilogram/daily with a 10-milligram decrease every week). Additionally, the patient underwent a renal biopsy, which demonstrated glomeruli necrosis with segmental sclerosis, hypercellularity with leukocyte infiltration, and hyaline deposits, which together were indicative of severe Grade IV lupus nephritis. He restarted his hydroxychloroquine and was also initiated on mycophenolate mofetil (360 milligrams twice daily) and tacrolimus (2 milligrams twice daily) for treatment of his lupus nephritis. At the patient’s outpatient hospital follow-up appointment, he was noted to have discontinued his hydroxychloroquine, mycophenolate mofetil, and tacrolimus. He was educated by his Rheumatologist on the importance of treatment of his SLE and lupus nephritis, and he was agreeable to restart his medications.

Several months following his second hospitalization, the patient continued to have intermittent adherence to his medications per outpatient documentation. His mycophenolate mofetil was transitioned to mycophenolic acid for reported gastrointestinal upset. Despite adjustments to his medications to mitigate side effects, outpatient labs revealed infection with Clostridium difficile, bicytopenia secondary to relapsed ES as well as worsening creatinine with life-threatening hyperkalemia. The patient was sent to the emergency department, where his hyperkalemia was promptly treated and he was admitted to the medical intensive care unit for acute renal failure. He also started oral fidaxomicin for the treatment of Clostridium difficile. While undergoing placement of a dialysis catheter, the patient developed seizure-like activity and hypoxic respiratory failure requiring intubation. A non-contrast CT of his head was concerning for an acute infarct of the left frontal lobe, so the patient was started on low-dose aspirin. Although the patient was given 5 days of methylprednisolone and restarted on hydroxychloroquine, the patient was also started on intravenous cyclophosphamide 500 milligrams according to the EURO-Lupus protocol (500 milligrams intravenous infusion every 2 weeks for 3 months) and plasmapheresis given the severity of his SLE and ES flares. Shortly after receiving cyclophosphamide, the patient developed tachycardia, significant elevation in troponins, and episodes of non-sustained ventricular tachycardia on telemetry. Emergent point-of-care ultrasound of the heart revealed a significantly decreased ejection fraction. Cardiology was consulted, who voiced concerns for the development of cardiogenic shock secondary to cyclophosphamide-induced myocarditis. The patient was taken for placement of veno-arterial extracorporeal membrane oxygenation (VA-ECMO) and additional hemodynamic support with a percutaneous ventricular assist device. While monitoring for cardiac recovery, the patient was noted to have a moderate pericardial effusion, suspected to be from his SLE, and underwent placement of a pericardial drain. The patient briefly recovered and was able to be weaned off VA-ECMO. Unfortunately, he started to develop hematemesis and rectal bleeding, which led to a significant drop in hemoglobin. The patient underwent a massive transfusion protocol (transfusion of blood products in a ratio of 1 unit of packed red blood cells to 1 unit of plasma to 1 unit of platelets). The Gastrointestinal Department was consulted, which deemed the patient to be too high risk to undergo endoscopy, so a pantoprazole drip was initiated. His rectal bleeding was believed to be due to a fulminant Clostridium difficile infection, so oral fidaxomicin was transitioned to intravenous eravacycline and rectal vancomycin. He again briefly improved and was started on total parenteral nutrition (TPN) given his prolonged hospital course and poor oral intake. He then developed ascites and hypotension, thus raising concern for abdominal compartment syndrome. He underwent paracentesis with removal of 5.5 liters and improvement in hypotension; however, his fluid cultures grew Candida lusitaniae, a rare yeast that often infects immunocompromised patients. He was started on intravenous anidulafungin. A CT angiography of the abdomen and pelvis was completed and revealed diffuse bowel wall edema with increased mucosal enhancement suggestive of hypo-perfusion state as well as a thrombosis of the left femoral vein. He could not be started on anticoagulation due to his recent severe gastrointestinal bleeding. Shortly thereafter, he developed severe persistent hypoglycemia despite aggressive attempts to supplement with intravenous glucose. The patient’s family opted to pursue hospice and he unfortunately passed away.

## Discussion

This case describes a young male patient who was newly diagnosed with SLE, which was complicated by AIHA and ITP. This presentation is consistent with secondary ES in the setting of SLE. This patient underwent extensive workup to confirm ES, as it is a diagnosis of exclusion. Other diagnoses that must be excluded are TTP, cold agglutinin disease, myelodysplastic syndromes, autoimmune diseases, and malignancy [6,7]. Initial investigation for ES should include a complete blood count, reticulocyte count, haptoglobin, lactate dehydrogenase, unconjugated bilirubin, and a direct antiglobulin test. To exclude other diagnoses or to determine the secondary nature of ES, studies such as peripheral blood smear, viral testing, serum protein electrophoresis and fixation, autoimmune antibodies, and CT chest, abdomen, and pelvis should be performed [6,7].

Since ES is a rare condition, there have been no large prospective or randomized trials to investigate optimal treatment strategies. Therefore, current treatment recommendations are extrapolated from the treatment of isolated ITP, AIHA, and AIN as well as from recently published expert consensus [7]. First-line therapy consists of full-dose corticosteroids (i.e. prednisone, 1 milligram per kilogram daily) followed by a taper, the addition of intravenous immunoglobulin (IVIG) for more severe disease, and transfusion support. Evans Syndrome can be resistant to initial treatment and has a high rate of relapse after discontinuation of first-line therapy. Therefore, it often requires the use of additional agents or multiple agents in combination. Second-line treatments include rituximab, thrombopoietin receptor agonists (TPO-RA), erythropoietin (EPO) stimulating agents, and granulocyte colony-stimulating factor (G-CSF) [6,7]. Rituximab has been shown to be a useful tool in treating isolated cytopenias, however, it has slightly lower response rates in ES. In one case series, the initial response rate to rituximab was 82% but dropped to 64% at one year [5]. In a study that investigated SLE-associated immune cytopenias, rituximab had an overall response rate of 50% in those patients with ES [8]. Ultimately, expert consensus recommends the use of rituximab in those patients without initial response to first-line therapy, at first relapse of disease, in those with prior thrombotic events, and in patients with antiphospholipid antibodies, rheumatologic diseases, or lymphoproliferative disorders [7]. Thrombopoietin receptor agonists can also be considered for platelet support in relapsed ES patients with immunodeficiencies, a history of severe infections, or associated AIN [7]. One small retrospective study evaluated patients with ES who receive treatment with TPO-RAs and noted improvement in platelet counts; however, ES patients did have a greater relapse with the discontinuation of TPO-RAs and an increase in high-grade (grades 3 and 4) thrombotic adverse events when compared to patients with isolated ITP [9]. Erythropoietin-stimulating agents are now strongly recommended in ES patients, especially those with inadequate reticulocytosis (reticulocyte count less than 150 x 109 cells per liter, or less than 250 x109 cells per liter if the hemoglobin is less than 8 grams per deciliter) [7]. This recommendation is supported by a study that looked at the use of recombinant EPO in those with isolated AIHA, which reported a median hemoglobin increase of 2 grams per deciliter in 70% of treated patients [10]. This group also conducted a phase 2 prospective study in patients with AIHA, including 6 patients with ES, and showed that recombinant EPO induced a significant hemoglobin response of 74% in 1 month and 91% at 12 months [11]. In ES patients with AIN, G-CSF may be used for patients during severe (grade 3 or 4) infections when absolute neutrophil count drops below 1000 cells per microliter. Prophylactic antibiotics, antivirals, and/or antifungals may be utilized in patients with persistent absolute neutrophil counts below 500 cells per microliter and who have had at least one high-grade (grade 3 or 4) infection per year [7].

In those patients with severe refractory ES, immunosuppressants (i.e., mycophenolate mofetil, sirolimus, azathioprine, or cyclophosphamide), splenectomy, and very rarely hematopoietic stem cell transplantation (HSCT) may be considered [6,7]. A small study investigating sirolimus in patients with relapsed and refractory warm AIHA or ES were treated with sirolimus for a median of 23 months and reported 57% of patients having achieved complete response [12]. However, immunosuppressants are only considered after multiple relapses of disease and may be more beneficial in patients with ES secondary to autoimmune disease [7]. Therapeutic plasma exchange (TPE) has also been utilized in patients with refractory or severe SLE with concomitant hematologic manifestations. In one retrospective database study, TPE was used to temporize treatment-resistant SLE patients with thrombocytopenia, hemolytic anemia, or ES [13]. Robust responses were seen in those SLE patients with hemolytic processes while SLE patients with thrombotic processes appeared to be less responsive to TPE [13]. In patients with severe refractory disease and steroid dependence, splenectomy may be considered. In a single-center retrospective study, splenectomy was found to have an initial response rate of 85.7%, however, it had a relapse rate of 42.8% within 1 year [14]. Based on expert consensus, splenectomy should be avoided in patients with ES with autoimmune disorders, the presence of antiphospholipid antibodies, and previous thrombotic events due to an increased risk of thrombosis following splenectomy [6,7]. Finally, HSCT is very rarely offered and is restricted to select patients who are refractory to multiple therapies [6]. Overall, it can be extremely challenging when treating ES patients.

ES can be a primary diagnosis or associated with another etiology. Systemic lupus erythematosus is the most common autoimmune disease ES is associated with [5]. In a study done by Costallat et al., it was found that ES often presents at the onset of SLE diagnosis or in the setting of active lupus flares, and this concomitant presentation was associated with more severe multisystemic disease [15]. There is limited investigation into the morbidity and mortality of primary and secondary ES, however, the few that have been done have shown overall poor clinical outcomes. Significant complications such as bleeding, thromboses, and infections often occur. In a case series by Fattizzo, 21% of the patients experienced a thrombotic episode and 33% of patients had at least one infectious episode, with 1 and 3 fatal events respectively [3]. Interestingly, this study also found that infections were more frequent in patients with secondary ES, particularly those with a positive ANA [3]. In another multicenter study that investigated outcomes of ES, they found that the median survival rate post-diagnosis was 7.2 years. Of note, there was a significant discrepancy between the survival rate between primary ES (median survival of 10.9 years) versus secondary ES (median survival of 1.7 years) [4]. This discrepancy was suspected to be due to 77.3% of the secondary ES cohort being in the setting of hematologic malignancies, which may confound survival rates. Unfortunately, a diagnosis of ES confers a poor prognosis.

## Conclusions

Evans Syndrome is a diagnosis of exclusion that can be difficult to treat and is associated with high morbidity and mortality. Given the complex nature of this disease, prompt diagnosis and treatment are imperative for these patients. In our case, the patient was fully worked up and diagnosed with ES within 10 days of his initial presentation. He was quickly initiated on first-line therapy for ES with corticosteroids and started on hydroxychloroquine for treatment of his SLE. Throughout his difficult clinical course, he was treated with multiple lines of therapy. Despite attempts from multiple providers to educate the patient about the importance of adherence to medical therapies, the patient continued to have difficulties taking his immunosuppressive medications due to intolerable side effects. The patient suffered multiple relapses of his SLE, with the last flare being complicated by thrombosis, infections, and severe bleeding that led to multiorgan failure. Unfortunately, the multisystem effects of his SLE and ES led to the premature death of the patient.
